# Eye irrigation as a first-line treatment and diagnostic method for emergency department patients who complain of ocular foreign bodies

**DOI:** 10.1038/s41598-021-02989-3

**Published:** 2021-12-03

**Authors:** Hung-Da Chou, Kuan-Jen Chen, Eugene Yu-Chuan Kang, Jui-Yen Lin, Po-Han Yeh, Yen-Ting Chen, Chi-Tung Cheng, Chi-Chun Lai, Wei-Chi Wu, Yih-Shiou Hwang, Ching-Hsi Hsiao

**Affiliations:** 1 Department of Ophthalmology, Chang Gung Memorial Hospital, Linkou Medical Center, No. 5, Fuxing St., Gueishan Dist., Taoyuan, 333 Taiwan; 2 College of Medicine, Chang Gung University, Taoyuan, Taiwan; 3 Department of Trauma and Emergency Surgery, Chang Gung Memorial Hospital, Linkou Chang Gung University, Taoyuan, Taiwan; 4 Department of Ophthalmology, Chang Gung Memorial Hospital, Keelung, Taiwan

**Keywords:** Conjunctival diseases, Corneal diseases, Trauma

## Abstract

This prospective study aimed at determine whether eye irrigation removes ocular foreign bodies (FBs) and whether ocular pain predicts FBs. Emergency department patients complaining of ocular FBs were enrolled. In the irrigation group (n = 52), pain was evaluated with a visual analog scale before and after irrigation, and the presence of FBs was determined under a slit-lamp. In the nonirrigation group (n = 27), the evaluations were performed upon arrival. The corneal FB retention rate was found significantly lower in the irrigation (13/52, 25%) than in the nonirrigation groups (13/27, 48%; *P* = 0.04). After irrigation, those without FBs had more patients experiencing pain reduction (67%) compared to those with retained FBs (46%; *P* = 0.14) and had a greater magnitude of change in pain score (mean ± SD, − 2.6 ± 2.7 vs. − 0.7 ± 1.4; *P* = 0.02). An improvement in ocular pain score ≥ 5 points after irrigation predicted the absence of FBs with a negative predictive value of 100%. Eye irrigation significantly lowered corneal FB retention; if ocular pain decreased considerably, the probability of retained FBs was low, making irrigation-associated pain score reduction a feasible diagnostic method to exclude FB retention without needing specialized ophthalmic examinations.

## Introduction

Eye-related problems are a common reason for emergency department (ED) visits, and the frequency of such visits is increasing^1^. In 2010, 1.5% of patients visiting the EDs in the United States received an ophthalmic principal diagnosis, and, between 2000 and 2015, 2–3% of patients discharged from the EDs in Taiwan received diagnoses of eye-related injuries^2,3^.

Among patients visiting EDs with ophthalmic primary complaints, corneal abrasions and foreign bodies (FBs) on the ocular surface are among the leading diagnoses, and these two diagnoses respectively represent approximately 13–14% and 8–15% of the complaints^4,5^. These patients probably visited the ED because of persistent ocular FB sensation and concerns that a FB remained inside the eye. However, a recent report from New York City revealed that the time from triage to completion of an ophthalmologic consultation for patients complaining of FB sensation was long: 174–263 min^5^.

One reason for the long wait is that, although ophthalmic problems are common, ED physicians are uncomfortable dealing with such problems. In the United Kingdom, two surveys conducted 10 years apart reported that 64–69% of senior house officers in EDs had little or no confidence in managing patients with ophthalmic complaints, despite improvements in their ophthalmic training programs^6,7^. Additionally, although 84% of EDs had slit-lamps available, 30% of ED physicians reported no confidence at all in using the instrument for examination^6^. Such a lack of confidence could lead to avoidable specialist consultations and thus entail long ED waits. In our hospital, where 24-h emergency eye service is available, 11.8% of all consultations in the ED were sent to ophthalmologists, which ranked first among all consultations^8^.

External eye irrigation is a standard protocol in the ED for treating ocular chemical injuries^9^. In our ED, after excluding open-globe injuries, physicians commonly order eye irrigation for patients complaining of ocular FBs. During the ophthalmologic consultations that follow, many patients report that their ocular pain greatly diminished after irrigation, and among such patients, retained FBs are uncommon.

On the basis of the above observations, we speculated that external eye irrigation could wash out ocular FBs and reduce ocular pain. Additionally, we assumed that after irrigation, an absence of retained FBs could be predicted from a patient’s considerably improvement in the subjective ocular pain sensation. If these theories tested to be true, eye irrigation could be considered as the first-line treatment and a diagnostic procedure which rendering further examinations with special ophthalmic instruments or techniques unnecessary for this common condition in EDs.

## Methods

### Patients

This prospective observational cohort study was conducted at a tertiary ED in Chang Gung Memorial Hospital, Linkou, Taiwan. The study was approved by the hospital’s institutional review board (No. 201600962A3C101) and adhered to the tenets of the Declaration of Helsinki. Written informed consent was obtained from all enrolled patients.

**Figure 1:**
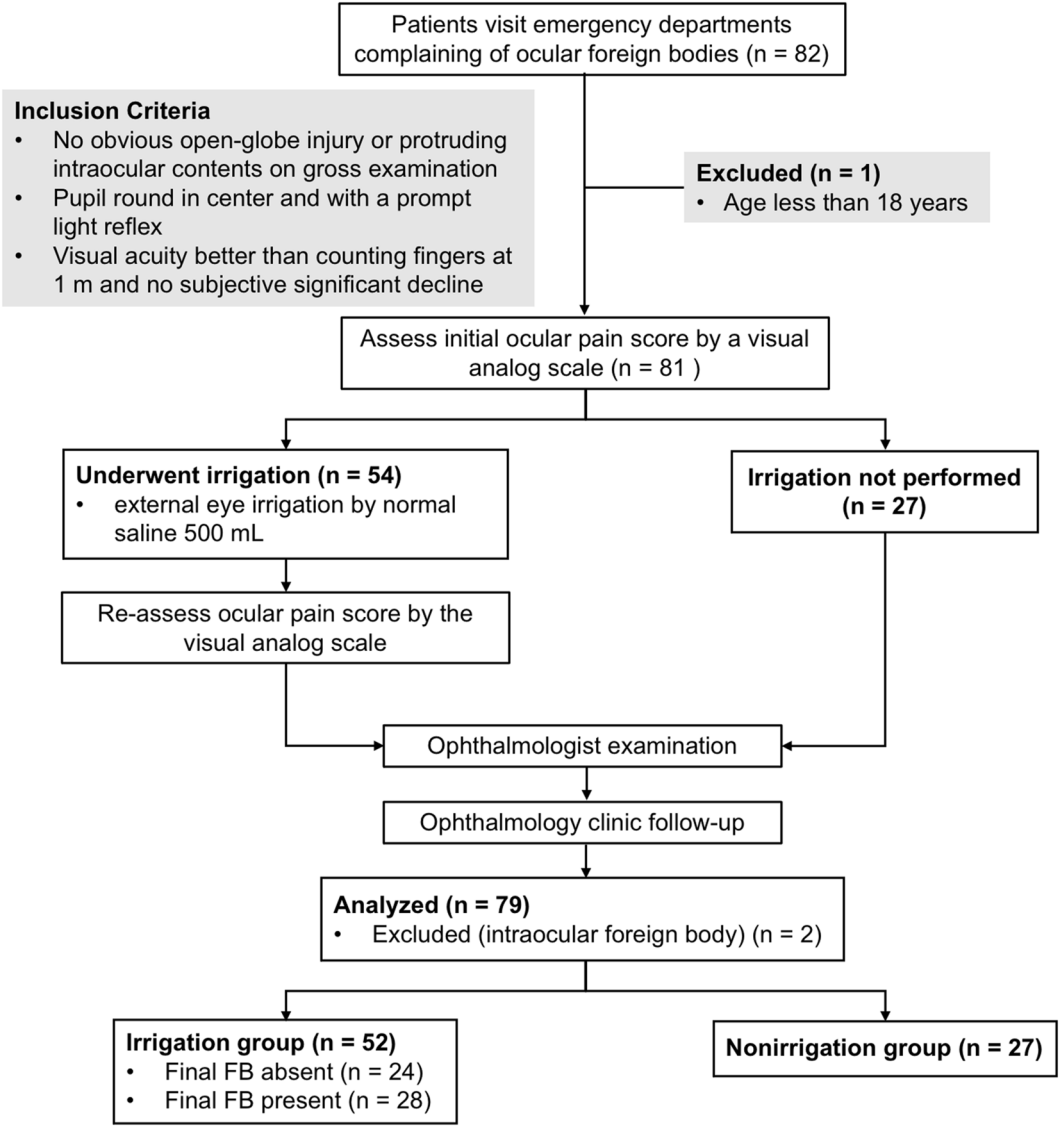
Study flowchart and patient grouping.

Patients who visited the ED between November 2016 and July 2017 complaining of a foreign body went into the eye, accompanied by persistent ocular discomfort were initially examined by an ED physician. The onset, duration, and symptoms of the eye discomfort were obtained, and basic ophthalmic and general medical history were documented. If both eyes were affected, each eye was separately evaluated. Patients who had no obvious open-globe injury or protruding intraocular content, had a round-in-center pupil with a prompt light reflex, could count fingers at 1 m, and experienced no considerable subjective decline in visual acuity were enrolled in this study. Patients who were younger than 18 years or could not complete a questionnaire were excluded (Fig. 1). The enrolled patients were allocated to the irrigation group in the first 3 months of the study, and their ophthalmic outcomes was reviewed at the end of month 3 to ensure no adverse event. Then, in the following 6 months, the enrolled patients were allocated to irrigation or nonirrigation group in alternate months.

### Ocular pain score measurements and external eye irrigation by ED staff

The ocular discomfort of the enrolled patients was quantified by an emergency physician or nurse using a visual analog scale with standardized questions (VAS; see Supplementary Fig. S1 online). After recording the VAS score, the patients in the irrigation group received irrigation with 500 mL of normal saline as follows: a bag of sterile normal saline connected to an intravenous infusion set without an intravenous catheter was suspended 1-m above the patient’s head on an intravenous drip hanger; the patient lay on the side of the affected eye. The ED nurse manually opened the affected eye and carefully performed external eye irrigation with the intravenous infusion line. Care was taken not to compress or rub the eye during the process. No analgesic eyedrops were given before or after irrigation. At least 5 min after irrigation, the same VAS was used to evaluate ocular pain, and a subsequent examination was arranged with an ophthalmologist. In the nonirrigation group, the patients went directly to an ophthalmologist after evaluating the VAS score.

### Ophthalmic examination by ophthalmologists

On the same day following managements in the ED, an on-call ophthalmologist surveyed the patient by asking the details of the incident, and an occupational questionnaire was completed if the patient reported that the injury occurred during work. Distant visual acuity was measured using a pinhole occluder or the patient’s own spectacles and a C-chart at 6 m. Slit-lamp biomicroscopy was used to detect and locate the FB, and fluorescein eye staining was performed to determine the degree of ocular surface injury. The maximal length and width of an epithelial defect were estimated under the slit-lamp, and the total epithelial defect area was calculated. For linear abrasions, if they presented as several abrasion lines within a focal area, the affected size was calculated using the length and width of the abrasion. If the abrasion was a single line, the length of the abrasion was measured, and the width was presumed to be 0.5 mm for size calculation. To quantify the degree of punctate abrasions, a punctate abrasion score (0–15), similar to the score used for dry eye evaluation, was used^10^. After the examination, the FB, if present, was carefully removed from the ocular surface.

### Management and follow-up

Upon confirmation of the FB being removed from the ocular surface, the patient was discharged, and one drop of chloramphenicol 0.25% (Sinphar, Yilan, Taiwan) was prescribed for application to the affected eye four times a day if the patient had only minimal ocular surface defects and did not regularly use contact lenses. An ophthalmology clinic follow-up was arranged within 1–3 days. If the epithelial defect area was larger than approximately one-sixth of the corneal area, the FB was present upon examination, or the patient regularly used contact lenses, one drop of levofloxacin 0.5% (Santen, Taipei, Taiwan) to be used four times a day, and tobramycin ointment (Alcon Cusi S.A., Barcelona, Spain) to be applied at night were prescribed, and an ophthalmology clinic follow-up was arranged within 24 h. The ophthalmic conditions in the follow-up clinics were collected from medical records, and patients were divided into a nonirrigation group and an irrigation group for comparison, and among the irrigation group, into FB retention and FB absent subgroups for analysis.

### Statistical analyses

The estimated sample size was calculated based on the preliminary results from the first 3 months of this study. The rate of finding a retained FB was assumed to be 70% without external eye irrigation, and irrigation was assumed to reduce the rate of retained FBs by 50%. Under the assumption that the irrigation group would be twice the size of the nonirrigation group, the estimated sample size was 46 and 23 for the irrigation and nonirrigation groups, respectively (alpha 5%, power 80%).

Data are presented as mean ± standard deviations. If both eyes were affected, only the eye with the higher initial pain score was included in the analysis. The normality of the distribution of continuous variables was tested with the Shapiro–Wilk test. Variables were compared using the *χ*^2^ test for categorical variables and independent *t*-test and Mann–Whitney *U* test for normally and non-normally distributed continuous variables, respectively. VAS scores before and after irrigation were compared using the Wilcoxon Signed Ranks test. Spearman’s Rank correlation coefficients were calculated. Receiver operating characteristic (ROC) curves were constructed using the sensitivity and specificity of the ocular pain scores for predicting the presence or absence of FBs after irrigation, and the area under the ROC curve (AUC) was analyzed. All statistical analyses were performed using SPSS Ver. 25 (IBM Corp., Armonk, NY). A two-sided *P* of < 0.05 was considered statistically significant.

### Ethics approval

The study was approved by the Institutional Review Board (No. 201600962A3C101) and adhered to the tenets of the Declaration of Helsinki.

## Results

### Clinical characteristics

After two patients with an intraocular FB discovered during ophthalmologist examination were excluded, 79 patients were analyzed. The mean ages were 44.8 and 44.0 years in the nonirrigation (n = 27) and irrigation (n = 52) groups, respectively, and the majority of the patients were male (Table 1). The ocular pain scores on arrival to the ED were similar in the nonirrigation (4.0 ± 2.8) and irrigation (4.4 ± 2.9) groups (*P* = 0.50).

**Table 1:** Demographics and clinical characteristics. *ED* emergency department, *LogMAR* logarithm of the minimum angle of resolution, *VAS* visual analog scale. ^a^Distant visual acuity measured using a pinhole occluder or the patient’s own spectacles and a C-chart at 6 m.

	Nonirrigation group	Irrigation group	*P* value
*n*	27	52	
Age, mean ± SD, y	44.8 ± 15.8	44.0 ± 13.7	0.82
Male, n (%)	20 (74)	43 (83)	0.37
Right eye, n (%)	12 (44)	26 (50)	0.64
Visual acuity, mean ± SD^a^, logMAR	0.0 ± 0.1	0.1 ± 0.1	0.27
Ocular pain score upon ED arrival, mean ± SD, VAS	4.0 ± 2.8	4.4 ± 2.9	0.50
**Self-reported features upon ED arrival, n (%)**			
Injured at work	15 (56)	30 (58)	0.86
Possibly metallic FB	11 (41)	20 (39)	0.84
High speed FB when it hit the eye	7 (26)	8 (35)	0.26
**Presence of foreign body upon ophthalmologist examination, n (%)**			
On the cornea	13 (48)	13 (25)	0.04
In the conjunctival sac	6 (26)	17 (33)	0.33
No foreign body found	9 (33)	24 (46)	0.27
**Corneal injury, mean ± SD**			
Epithelial defect area, mm^2^	0.5 ± 1.2	0.8 ± 3.0	0.60
Linear abrasion area, mm^2^	0.9 ± 2.5	2.5 ± 9.5	0.76
Punctate abrasion score	1.7 ± 2.4	2.6 ± 2.7	0.10
**Conjunctival injury, mean ± SD**			
Epithelial defect area, mm^2^	0.2 ± 1.2	0.4 ± 2.2	0.96
Linear abrasion area, mm^2^	0.1 ± 0.3	0.0 ± 0.0	0.17
Punctate abrasion score	0.0 ± 0.0	0.7 ± 1.8	0.02

### Ocular FB retention rate and injury scores

The presence of the FB and ocular surface injury were evaluated by an ophthalmologist; the evaluation was performed immediately for the nonirrigation group and after eye irrigation in the irrigation group. FB retention in the irrigation group was significantly lower (13 of 52 eyes, 25%) than that in the nonirrigation group (13 of 27 eyes, 48%; *P* = 0.04). By contrast, the proportions of eyes with FBs retained in the conjunctival sac were similar in the two groups (33% and 26% in the irrigation and nonirrigation groups, respectively; *P* = 0.33). In total, FBs were absent from 33% and 46% of eyes in the nonirrigation and irrigation group, respectively (*P* = 0.27; Table 1).

The ocular surface injury scores for the cornea and conjunctiva were comparable between the groups, except for the conjunctival punctate abrasion score, which was significantly higher in the irrigation group than in the nonirrigation group (*P* = 0.02). No significant correlation was discovered between the ocular surface injury and ocular pain scores.

Of the 52 patients who received irrigation, 9 (17.3%) returned to the ophthalmology clinic as scheduled. Among these patients, one had a retained subconjunctival sand-like FB without active inflammation. The other eight patients had recovered, and none of the patients had signs of infection. Another 16 patients (30.8%) returned to other clinics in our hospital without revisiting the ophthalmic clinic. We presumed that these patients had recovered well since no ocular complaints were documented and no further ophthalmic clinic appointments were arranged.

### Change in ocular pain score and prediction of retained FBs

**Figure 2:**
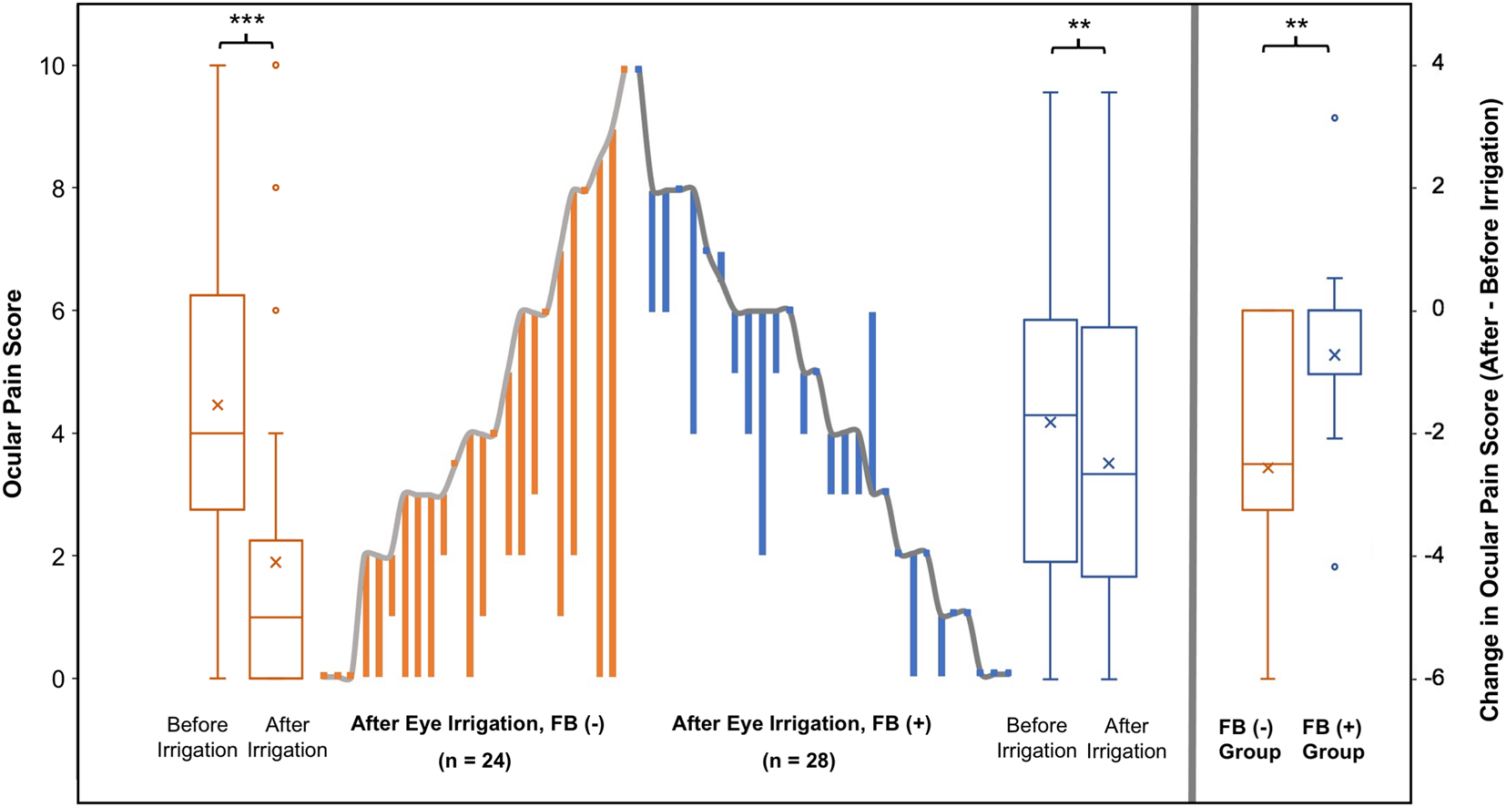
Change in ocular pain scores after external eye irrigation. The central parallel line plot depicts each patient’s pain score before and after irrigation, and the box plots show the distribution of the scores in each group. Half of the patients in the FB (+) group (blue) showed no improvement in ocular pain after irrigation, and the mean change in pain score was significantly less in this group of patients. (*FB* foreign body. The presence of FB was assessed after irrigation. **P < 0.01; ***P < 0.001).

For the 52 patients in the irrigation group, ocular discomfort was re-evaluated after irrigation. We further divided these patients into a FB (−) (n = 24) and a FB (+) (n = 28) group based on whether a retained FB was found after irrigation (Fig. 2). In the FB (−) and FB (+) groups, their ocular pain score significantly decreased from 4.5 ± 2.9 and 4.4 ± 2.9 before irrigation, to 1.9 ± 2.7 and 3.7 ± 2.7 after irrigation (*P* < 0.001 and *P* = 0.008, respectively). Notably, patients in the FB (−) group had a significantly greater change in pain score when compared to the FB (+) group (− 2.6 ± 2.7 vs. − 0.7 ± 1.4; *P* = 0.008).

**Figure 3:**
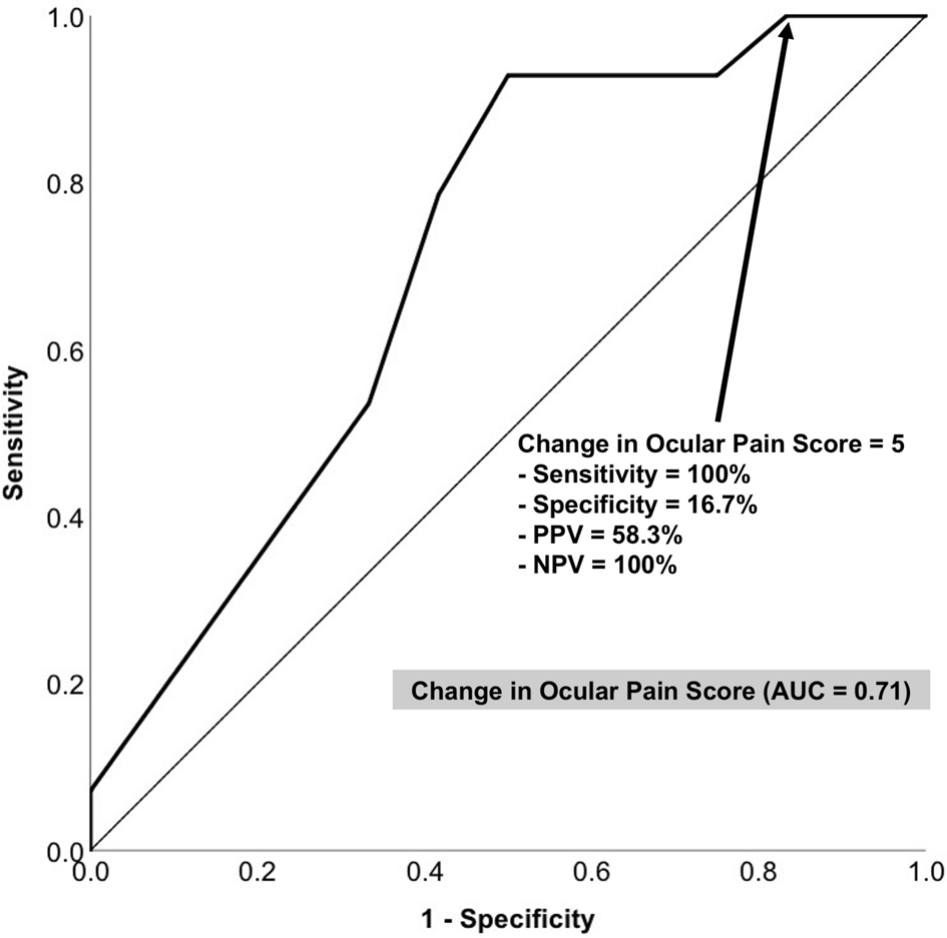
Receiver operating characteristic (ROC) curve of using the change in ocular pain score after external eye irrigation to discriminate the presence versus the absence of retained ocular foreign bodies. The thin line is a reference line (*AUC* area under the ROC curve, *NPV* negative predictive value, *PPV* positive predictive value).

Using the ROC curve and the AUC, the diagnostic ability of using the change in the ocular pain score after irrigation to detect a retained FB was analyzed, which showed that the change in ocular pain score had an AUC of 0.71 (95% confidence interval (CI), 0.56–0.85; *P* = 0.011) in diagnosing FB retention (Fig. 3). Further analysis of the coordinates of the ROC curve demonstrated a 100% sensitivity and 16.7% specificity for detecting retained FBs when the ocular pain score decreased less than 5 points after irrigation, with a positive predictive value of 58.3% and a negative predictive value of 100%. That is, if the ocular pain markedly improved after irrigation (pain score decreased 5 points or more, i.e., diagnostic test negative), the probability of a patient not having an FB was 100%. However, when the ocular pain did not change much after irrigation (pain score decreased less than 5 points after irrigation, i.e., diagnostic test positive), the probability of a retained FB was nearly 60%.

### Self-reported factors related to retained FBs

Whether self-reported factors could be used to predict the retention of FBs in the eye was investigated. When a patient reported still feeling an FB, that the FB was possibly metallic in nature, or that the incident had occurred during work, the relative risks of a retained FB were 1.73 (95% CI 1.13–2.66), 1.53 (95% CI 1.78–3.91), and 1.82 (95% CI 1.15–2.89), respectively (Table 2). Whether the FB had impacted the eye at a high speed was not significantly associated with the probability of FB retention.

**Table 2:** Self-reported risk factors related to retained ocular foreign bodies (FBs). The risk factors were based on the report by patients (n = 79) upon emergency department arrival. The presence of FBs was evaluated after external eye irrigation in 52 of the 79 subjects. *CI* confidence interval.

	Relative risk	95% CI	*P* value
Injured at work	1.82	1.15–2.89	0.004
Subjective feeling that the FB was definitely present in the eye	1.73	1.13–2.66	0.006
Possibly metallic FB	1.53	1.78–3.91	< 0.0001
High speed FB when it hit the eye	1.11	0.67–1.85	0.669

### Results of occupation-related questionnaire

Of the 79 patients, 45 (57.0%) reported that the incident had occurred at work. A majority of respondents (32 of 45, 71.1%) were metal workers or mechanics (Table 3). Although 42% of the patients injured at work responded that they had experienced similar incidents and 62% reported that protective goggles were available at their workplace, only 13% of patients wore goggles. Nearly half (42%) answered that they felt protective goggles were unnecessary despite 42% receiving educational courses on eye protection from their employers. Another 22% reported not wearing goggles because goggles obstructed their view.

**Table 3:** Occupation-related questionnaire responded by patients who were injured at work. *FB* foreign body. ^a^Valid n = 10. ^b^Valid n = 7 in the work-related injury subgroup and 5 in the metal workers and mechanics subgroup.

	Work-related injury subgroup
*n*	45
**Occupation, n (%)**	
Metal worker	19 (42)
Mechanic	13 (29)
Others	13 (29)
**Wears protective goggles during work, n (%)**	
Yes	6 (13)
No	36 (80)
Not answered	3 (7)
**Reasons not wearing protective goggles, n (%)**	
Discomfort during wear	4 (9)
Blocks view	10 (22)
Feel not necessary	19 (42)
Not answered	12 (27)
**Prior FB incident occurred during work, n (%)**	
Yes	19 (42)
No	24 (53)
Not answered	2 (4)
Number of FB incident occurred during work, mean (range)^a^	11.1 (1–50)
**Have taken leave from work due to FB incident, n (%)**	
Yes	4 (9)
No	36 (80)
Not answered	5 (11)
Duration of leave from work due to FB incident, h, mean ± SD^b^	18.7 (0–80)
**Employer have provided educational courses on eye protection, n (%)**	
Yes	19 (42)
No	23 (51)
Not answered	3 (7)
**Employer provides protective googles, n (%)**	
Yes	28 (62)
No	14 (31)
Not answered	3 (7)

## Discussion

In the present prospective cohort study, we determined that external eye irrigation significantly reduced ocular pain and the probability of a retained corneal FB in patients visiting EDs with complaints of ocular FBs. In addition, if a patient’s subjective ocular pain greatly diminished after irrigation, the likelihood of a retained FB was very low.

The removal of an embedded corneal FB entails risks and requires certain techniques and training. To avoid iatrogenic corneal perforation, clear slit-lamp visualization of the location and depth of the FB and careful removal under magnification are advised^11^. However, according to a survey conducted in Ireland, nearly half of ED physicians removed ocular FBs without the aid of a slit-lamp, whereas nearly three-quarters of EDs had them available^12^. Lack of training was reported to be the primary reason for not utilizing slit-lamps^12^. Accordingly, simulated eye models have been created to assist with slit-lamp-assisted FB removal training^13,14^.

In the present study, the lower rate of corneal FBs in the irrigation group (25%, vs. 48% in the nonirrigation group) indicated that some FBs were probably not embedded deeply in the cornea and could therefore be removed through gentle irrigation. ED nurses experienced in performing eye irrigation for chemical burns can learn this procedure easily with minimal additional training. However, the relatively high incidence of FBs in the conjunctival sac (33% in the irrigation group vs. 26% in the nonirrigation group) suggested that some of the dislodged corneal FBs might have ended up in the fornix. Compared with removal of embedded corneal FBs, removal of conjunctival FBs is associated with a lower risk and required skill level.

The cornea is densely innervated and among the most sensitive tissues^15^. Studies have attempted to quantify subjective eye discomfort in dry eye disease. However, such discomfort is affected by conditions including ocular surface diseases^16^, inflammation^17^, neuropathic factors^18^, and nonocular discomfort^19^. The purely mechanical stress encountered by patients with ocular FBs differs from the multifactorial dry eye disease. Therefore, instead of using dry eye disease evaluation scales, we used a combination of general pain assessment scales including 11-point Box Scale^20^, 5-category scale^21^, and Wong–Baker FACES Pain Rating Scale^22^ as our ocular discomfort measurement tool. These scales have been widely validated and are the gold standards for pain evaluation^20–23^. Nevertheless, the perceived magnitude of discomfort greatly varies between individuals and is affected by factors such as sex and antidepressant use^24,25^. In the present study, we initially tried to use the absolute mean pain score to predict FBs. Unsurprisingly, the mean score had a low discriminatory power (AUC = 47.6%). Instead, we used the changes in pain score after irrigation to minimize the interindividual differences and achieved a much higher AUC of 71%. Additionally, a cutoff of 5 points for the change in ocular pain score resulted in a high sensitivity (100%) in detecting retained FBs. Thus, the irrigation-related change in the ocular pain score is a simple and acquirable factor for predicting ocular FB retention.

Although studies have reported that 14–33% of the FBs removed from corneas exhibited positive cultures, 95% and 90% of the isolated microorganisms were sensitive to chloramphenicol and ciprofloxacin, respectively^26,27^. In a rural setting with nearly 300 participants, prophylactic chloramphenicol ointment prescribed within 18 h after corneal abrasion resulted in no corneal ulcers^28^. In addition, after noncomplicated corneal FB removal, only 1.7% of eyes exhibited infectious keratitis after treatment with prophylactic fourth-generation fluoroquinolone eye drops, and the development of an infection could be alarmed by the subjective worsening of ocular symptoms^29^. In the present study, we prescribed chloramphenicol for noncomplicated cases and levofloxacin drops for large abrasions, patients who wore contact lenses, and FBs retained after irrigation. None of the patients in the irrigation group who returned to the clinic developed infectious complications, and their ocular surface injuries all resolved. The above studies and our results suggest that the chances of developing infection under prophylactic antibiotics in abrasion-related ocular surface injuries are low and that external eye irrigation did not compromise outcomes.

The present study has several limitations. Two patients who complained of bilateral eye discomfort were included, and although we enrolled only the subjectively more painful eye, the sensation in the excluded eye might have affected the pain score of the study eye. Our use of the changes in each patient’s own pain score as the diagnostic factor could have minimized such an impact, but the results may nevertheless be inapplicable to patients affected bilaterally. Another two patients who underwent irrigation were diagnosed with intraocular FBs. They passed the initial open-globe screenings because their entry wounds were small, noncentralized, and self-sealed. Nonetheless, we used sterile saline for irrigation and cautiously avoided applying pressure to the globe. Because the entry wounds were sealed, the risks of intraocular infection and content protrusion should be low. In addition, the ocular pain score was unchanged after irrigation in these patients, which had warranted ophthalmologist consultation. Finally, the FBs were not confirmed by an ophthalmologist with a slit-lamp both before irrigation because the examination itself might affect the location of the FB, especially if the eyelid were to be everted, and this could alter the outcomes of irrigation.

**Figure 4:**
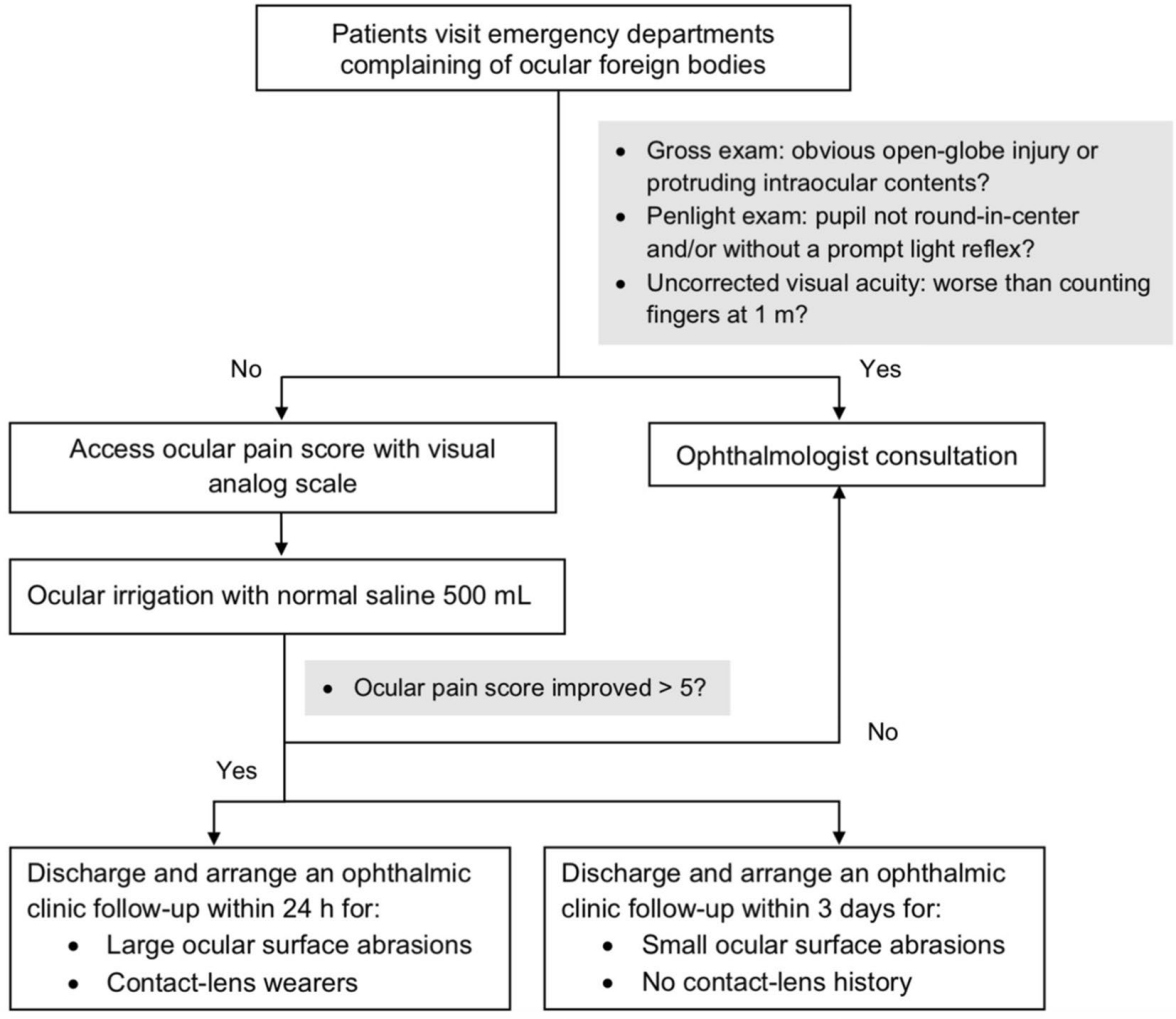
Proposed flowchart for patients who complain of ocular foreign bodies.

On the basis of our results, we propose a flowchart for patients with complaints of ocular FBs (Fig. 4). After open-globe injuries are excluded through basic examination, prompt eye irrigation is suggested to ease discomfort and remove corneal FBs. Changes in pain score after irrigation serve as a simple diagnostic indicator of FB retention. The flowchart can be used for instrument-free evaluation and may reduce the need for ophthalmologist consultation; this is especially beneficial in remote areas with few ophthalmologists as well as during the COVID-19 pandemic. However, further randomized controlled trials are warranted to confirm the results.

## Supplementary Information

Supplementary Figure S1.

## Data Availability

Data are available on reasonable request.
