# Supplementary figures and images for: Eye irrigation as a first-line treatment and diagnostic method for emergency department patients who complain of ocular foreign bodies
Source: Sci Rep. 2021 Dec 3;11:23386. doi: 10.1038/s41598-021-02989-3 (PMC8642417; doi:10.1038/s41598-021-02989-3)

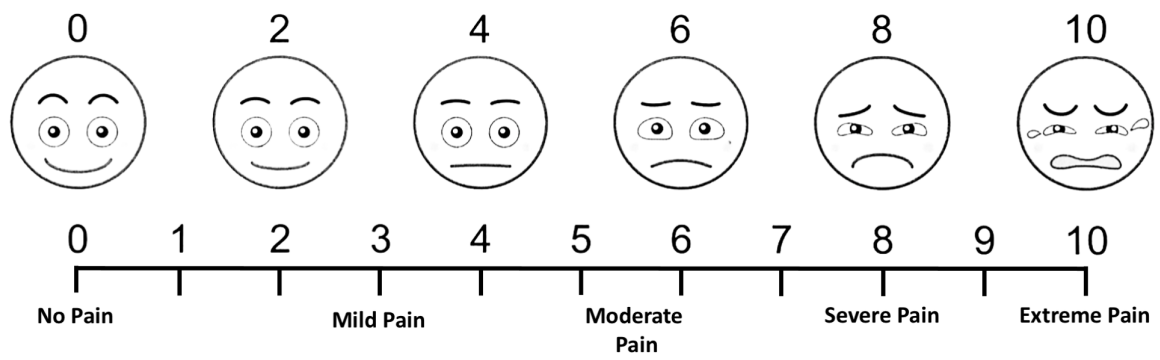

**Supplementary Figure S1.** Visual analog scale for the evaluation of ocular pain score.

Supplement: Supplementary file 1 — Supplementary Figure S1. [file 41598_2021_2989_MOESM1_ESM.pdf]
